# History of Medical Education and Institutions in the United States, Etc., with a Chapter on the Present Condition and Wants of the Profession, Etc.

**Published:** 1851-07

**Authors:** 


*History of Medical Education and Institutions in the United States, etc., with a Chapter on the present Condition and Wants of the Profession, etc.* By N. S. Davis, M. D., Prof. of the principles and practice of Med. in Rush Med. College, etc., etc. Chicago. S. C. Griggs & Co. 1851. 12 mo., pp. 228.

This work has been severely criticised by several of our contemporaries, and, in a late No. of the North Western Med. and Surg. Journal, the author appears in its defence. The history appeared originally in several numbers of the Journal just named, but, as a separate publication, has not been received by us until quite lately. This is the reason of our not having the work before. The chief source of critical animadversion is, that, as a history it is imperfect and inaccurate, particularly as respects the past literature of medicine in the United States. In answer to this objection, the author states that the work does not purport to be a history of medicine, or of medical literature, but of medical education, and institutions, in proof of which he refers to the title. It should not certainly be demanded of Dr. D. to furnish historical facts without the scope of the bibliographical plan which he adopts. In authorship, as in other matters, a person is only bound to do what he promises or undertakes to do. This plea is not valid, however, as to the charge of incorrectness in what are presented as facts. The inaccuracy especially complained of is contained in the following statement:—"I have not been able to find a single volume on any branch of medical science or practice, published by an American physician during the first twenty years after the close of the revolutionary war." Now, it is true that during this period several publications of more or less value appeared. The assertion is therefore incorrect, and does injustice to American medical literature. It is an unfortunate statement, but doubtless made honestly, in accordance with the information of the author at the time the paragraph was written. We would not condemn the book *in toto* on this account. We cannot but think, however, that it would have been better at once to concede the mistake, and endeavor to correct, rather than to defend it.

Nearly one-third of the volume is devoted to "the present condition and wants of the profession, and the remedies for those wants." The propositions contained in the following extract embody the views advocated in this portion of the work. We give the extract without comments at this time, for want of space:

1st. Extend their lecture terms to ten months, instead of four; divide it into three sections, with the medical sciences grouped into twelve divisions or chairs, four for each section of the term; and requiring four hours of each day to be occupied in regular lectures, and two in special attention to the study of practical anatomy, healthy and morbid, and to clinical medicine and surgery, with their appendages, including, of course, physical diagnosis, etc.

2d. Have the several chairs so arranged in each section of the term, that students should be required to attend one section only each year of their studies, the section for the last year embracing the branches more especially practical, such as practical medicine, surgery, midwifery, etc.

3d. Instead of the present system of charges, embrace the whole expense of attendance on any one section of the general term, except a charge for anatomical subjects and dissecting ticket, in an initiatory or matriculating fee of twenty-five dollars, payable always in advance—they would immediately induce a much larger proportion of students of medicine to attend lectures—they would make their medical education far more thorough, systematic, and practical, thereby greatly benefiting both the profession and the community; and they would rapidly concentrate in their own halls the whole patronage of the profession, and thereby benefit themselves and do much to destroy the petty college competition which is now so rife throughout the country. And if the sum now annually lost to the colleges, by giving students credit, and making deductions in special cases, should be added to that which would be paid by the additional number who would resort to these institutions, it would more than counterbalance the loss by a reduction of fees. Indeed, if the number of students resorting to the colleges should remain the same as at present, (four thousand five hundred), the charge of twenty-five dollars each would give one hundred and twelve thousand five hundred dollars annually, a sum sufficient to pay two hundred professors an average annual salary of five hundred dollars each, and leave twelve thousand five hundred dollars to defray the ordinary college expenses. These sums, taken in connection with the increased division of labor, in making twelve instead of seven chairs in each school, and the concentration of the whole or a much smaller number of schools properly located, would make stations in the colleges abundantly sought after, and as well paid as any other positions in the profession. And if, in connection with such a system of teaching, a complete and permanent system of social organization, requiring a proper preliminary education, and providing for an efficient board of medical examiners in each state, as already pointed out, was adopted, the medical profession of our country, numbering, as it now does in its ranks, many of the most eminent and learned men on the continent, would, as a whole, speedily assume a more elevated position, and occupy a much wider field of usefulness. In regard to the objection, that cheapening medical education would only increase the number of those who would crowd into the profession, I have simply to remark, that the numbers should be restricted by adding to the standard of requirements, instead of increasing the exactions on the student's pockets—the latter being both injurious and anti-republican in its tendencies.

